# Retraction: *PAR-2* promotes cell proliferation, migration and invasion through activating PI3K/AKT signaling pathway in oral squamous cell carcinoma

**DOI:** 10.1042/BSR-2018-2476_RET

**Published:** 2024-07-30

**Keywords:** Cell proliferation, Invasion, Migration, Oral squamous cell carcinoma, PAR-2, PI3K/AKT pathway

This Retraction follows an Expression of Concern relating to this article previously published by Portland Press. This article is being retracted from *Bioscience Reports* at the request of the Editor-in-Chief and the Editorial Board following receipt of a notification from a reader, alerting the Editorial Board to similarities in Western blot protein bands in Figure 6 Par-AP+DMSO group for PI3K, p-pI3K, and p-Akt. Additionally, the Editorial Office has identified similarities between protein bands of Figure 6 PAR-AP+LY294002 group for PI3K, p-PI3K, p-Akt, and PAR-AP+MK2206 p-Akt, and separately similarities within Figure 5A between the 200 µM PI3K and 0 µM AKT protein bands.

The Editorial Office was contacted by the first author Kai-Liang Tang from an email address that differed to the one provided at submission, requesting to withdraw the article after publication as the senior author Shi-Jiang Xiong was not fully informed of the submission or publication. The authors have since been contacted with regards to the retraction and have not responded to the Journal's queries or the concerns raised. Given the concerns surrounding the scientific validity and authorship of this paper, the Editorial Board stand by the decision to retract the article.

